# SnoReport 2.0: new features and a refined Support Vector Machine to improve snoRNA identification

**DOI:** 10.1186/s12859-016-1345-6

**Published:** 2016-12-15

**Authors:** João Victor de Araujo Oliveira, Fabrizio Costa, Rolf Backofen, Peter Florian Stadler, Maria Emília Machado Telles Walter, Jana Hertel

**Affiliations:** 1 Department of Computer Science, University of Brasilia, Brasília, BR-70910-900 Brazil; 2 Bioinformatics Group, Department of Computer Science, Albert-Ludwigs-University Freiburg, Georges-Köhler-Allee 106, Freiburg, 79110 Germany; 3 Bioinformatics Group, Department of Computer Science, and Interdisciplinary Center for Bioinformatics, University of Leipzig, Haertelstraße 16-18, Leipzig, D-04107 Germany; 4 German Centre for Integrative Biodiversity Research (iDiv), Halle-Jena-Leipzig, Germany; 5 Institute for Theoretical Chemistry, University of Vienna, Währingerstraße 17, Vienna, A-1090 Austria; 6 Center for non-coding RNA in Technology and Health, University of Copenhagen, Grønnegårdsvej 3, Frederiksberg, DK-1870 Denmark; 7 Max Planck Institute for Mathematics in the Sciences, Inselstraße 22, Leipzig, D-04103 Germany; 8 RNomics Group, Fraunhofer Institut for Cell Therapy and Immunology, Perlickstraße 1, Leipzig, D-04103 Germany; 9 Santa Fe Institute, 1399 Hyde Park Rd., Santa Fe, NM87501 USA; 10 Young Investigators Group Bioinformatics & Transcriptomics, Helmholtz Centre for Environmental Research – UFZ, Permoserstraße 15, Leipzig, D-04318 Germany

**Keywords:** Non-coding RNA, snoRNA, C/D box snoRNA, H/ACA box snoRNA, Machine learning, Support Vector Machine (SVM)

## Abstract

**Background:**

snoReport uses RNA secondary structure prediction combined with machine learning as the basis to identify the two main classes of small nucleolar RNAs, the box H/ACA snoRNAs and the box C/D snoRNAs. Here, we present snoReport 2.0, which substantially improves and extends in the original method by: extracting new features for both box C/D and H/ACA box snoRNAs; developing a more sophisticated technique in the SVM training phase with recent data from vertebrate organisms and a careful choice of the SVM parameters *C* and *γ*; and using updated versions of tools and databases used for the construction of the original version of snoReport. To validate the new version and to demonstrate its improved performance, we tested snoReport 2.0 in different organisms.

**Results:**

Results of the training and test phases of boxes H/ACA and C/D snoRNAs, in both versions of snoReport, are discussed. Validation on real data was performed to evaluate the predictions of snoReport 2.0. Our program was applied to a set of previously annotated sequences, some of them experimentally confirmed, of humans, nematodes, drosophilids, platypus, chickens and leishmania. We significantly improved the predictions for vertebrates, since the training phase used information of these organisms, but H/ACA box snoRNAs identification was improved for the other ones.

**Conclusion:**

We presented snoReport 2.0, to predict H/ACA box and C/D box snoRNAs, an efficient method to find true positives and avoid false positives in vertebrate organisms. H/ACA box snoRNA classifier showed an F-score of 93 % (an improvement of 10 % regarding the previous version), while C/D box snoRNA classifier, an F-Score of 94 % (improvement of 14 %). Besides, both classifiers exhibited performance measures above 90 %. These results show that snoReport 2.0 avoid false positives and false negatives, allowing to predict snoRNAs with high quality. In the validation phase, snoReport 2.0 predicted 67.43 % of vertebrate organisms for both classes. For Nematodes and Drosophilids, 69 % and 76.67 %, for H/ACA box snoRNAs were predicted, respectively, showing that snoReport 2.0 is good to identify snoRNAs in vertebrates and also H/ACA box snoRNAs in invertebrates organisms.

**Electronic supplementary material:**

The online version of this article (doi:10.1186/s12859-016-1345-6) contains supplementary material, which is available to authorized users.

## Background

Non-coding RNA genes (ncRNA genes) play important roles in the cell, e.g., structural, catalytic and regulatory functions [1, 2]. The study ncRNAs remains challenging, because laboratory experiments to confirm functions performed by one ncRNA are difficult to be performed, and many distinct computational methods find different results to identify and classify ncRNAs. One key problem is that ncRNA functions are closely associated to their spatial (secondary) structures, which prevents the use of methods to predict protein coding genes based only on their nucleotide sequences (primary structures).

Identification of ncRNAs have been developed for a variety of organisms [3–6], with the objective of constructing sets of different classes of ncRNAs. In particular, snoRNAs [7] are 60 to 300 nt ncRNAs, classified based on their characteristic sequence elements, called *boxes*, in two main classes: H/ACA box snoRNAs and C/D box snoRNAs. In humans [8], snoRNAs are usually found in intronic regions where, after splicing reaction, they escape from degradation by forming a protein complex [7]. Usually snoRNAs have a short stretch of sequence complementary to target RNAs, like rRNAs, tRNAs and snRNAs, performing chemical modifications on them. C/D box snoRNAs contains fibrillarin that promotes the 2’O-methylation on target RNAs, while H/ACA box snoRNAs contains dyskerin that catalyzes the conversion of uridine to pseudouridine [7, 9].

**Fig. 1:**
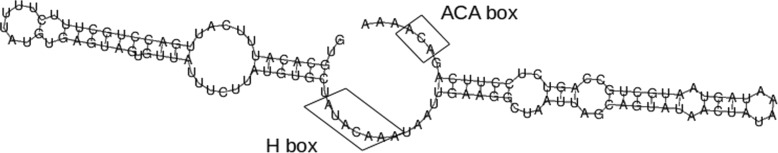
Example of *H/ACA box* snoRNA

H/ACA box snoRNA and C/D box snoRNA have distinct secondary structures. H/ACA box snoRNAs are formed by a double hairpin loop structure with two short-single stranded regions containing box H (ANANNA), located between the two hairpins loops, and box ACA (ACA) followed by 3 nt upstream the 3’ end. The hairpin loops have bulges, or recognition loops, which form the antisense element for target RNAs. Normally the first unpaired nucleotide inside the recognition loop is an uridine located 13–16 nt before the H and ACA boxes [7, 10, 11]. Figure 1 shows a schematic secondary structure of H/ACA box snoRNA.

**Fig. 2:**
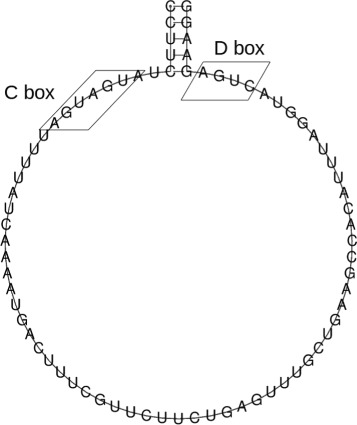
Example of *C/D box* snoRNA

C/D box snoRNAs are formed by two conserved boxes C (RUGAUGA, where R is a purine) and D (CUGA) near their 5’ and 3’ ends, separated by a short stem (3–10 nt). Inside the loop between C and D boxes, usually there are imperfect copies of C and D boxes, called C’ and D’. Normally the antisense element is located 5 nt upstream C’ and D’ boxes. Figure 2 shows a schematic secondary structure of a C/D box snoRNA.

SnoReport [9] is a tool that identifies the two main classes of snoRNAs in single sequences, using a combination of secondary structure prediction and machine learning. In contrast to previous methods for snoRNA identification (except snoSeeker [10]), snoReport prediction does not use information of putative target sites within ribosomal or spliceosomal RNA (this information can dramatically improve identification sensibility and specificity). However, many orphan snoRNAs have been discovered with the snoReport approach. The targets of orphan snoRNAs are not known, consequently such genes would be missed by target depending on the identification method [9, 12]. Beyond this, some snoRNAs are shown to target specific mRNAs, suggesting other functions, e.g., interference with A-to-I editing [7, 9, 12–14]. In order to identify C/D box and H/ACA box snoRNAs, snoReport uses position-specific weighted matrices (PWM’s) to identify boxes, together with a set of restrictions related to the secondary structure prediction, usually, restrictions about distance between regions of the secondary structure, and whether it forms the hairpins for H/ACA box snoRNAs, or the loop for a C/D box snoRNA.

SnoReport produced good results. In the test phase, snoReport presented 96 % of sensitivity and 91 % of specificity for the C/D box snoRNA classification, while for H/ACA box snoRNAs, it has shown 78 % of sensitivity and 89 % of specificity. However, snoReport has been trained on almost exclusively mammalian sequences, having used some default parameters for the Support Vector Machine (SVM) classifier. To date, many new sequences of snoRNAs for different vertebrate organisms have been identified, and experimentally confirmed. Furthermore, many tools and databases used to build snoReport have been improved. This suggests that snoReport has to be updated, in order to use new data and refined machine learning techniques to improve its performance.

We improved snoReport, by extracting new features for both box C/D and H/ACA box snoRNAs, developing a more sophisticated technique in the SVM training phase (with recent data from vertebrate organisms and a different approach to refine the *C* and *γ* SVM parameters), and using new versions of the tools and databases previously taken to build snoReport. To validate this new version of snoReport, we tested it in different organisms. These experiments have shown a very good performance.

This text is organized as follows. In the next section, we describe the methods used for building the new version of snoReport, particularly, data sources and the new workflow, besides the new features and details of the training phase. After, we show the results obtained by the new version of snoReport with different species of organisms. Following, we discuss these results. Finally, we conclude and suggest future work.

## Methods

First, data sources, software components, and the workflow used to build the new snoReport are described. Next, the new attributes for boxes H/ACA and C/D snoRNAs used in the SVM classifier are shown.

### Data sources

In snoReport, two datasets were used for the training and testing phases: positive samples and negative samples. The positive sample set was composed of H/ACA box and C/D box snoRNAs, while the negative one was obtained from a dinucleotide shuffling procedure executed in the positive samples with the EDeN [15] library.

The positive sequences from each class of snoRNAs were divided in two datasets, to be used in the learning process. In order to avoid overfitting, these datasets were created such that very similar sequences would not be stored in different datasets. First, we clustered the sequences using ClustalW [16] with criterion *nucleotide similarity*, which generated 157 clusters for C/D box snoRNA and 101 clusters for H/ACA box snoRNA. After, 10 sequences from distinct vertebrates organisms were extracted from each cluster, noting that clusters containing less than 10 sequences were discarded. Therefore, a consensus sequence from each cluster was obtained with ClustalW and Cons (for EMBOSS [17]), and these sequences were used to generate a distance tree, with the neighbour-joining method [18] from ClustalW2 - phylogeny [19]. The next step was to divide this distance tree in two parts, which allowed to create the two datasets containing similar sequences. The generated trees of C/D box snoRNA and H/ACA box snoRNA clusters can be viewed on Additional file 1.

Table 1 shows the number of sequences of each dataset.

**Table 1:** Number of sequences of Datasets 1 and 2 of both C/D box and H/ACA box snoRNAs

	Dataset 1	Dataset 2
C/D box snoRNAs	750	520
H/ACA box snoRNAs	490	420

**Fig. 3:**
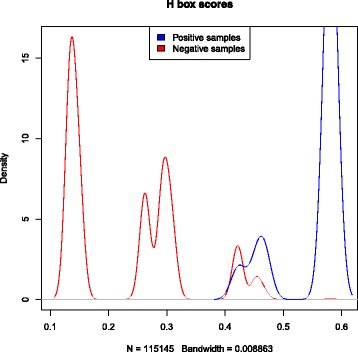
Density plot of *H box* PWM-based scores

**Fig. 4:**
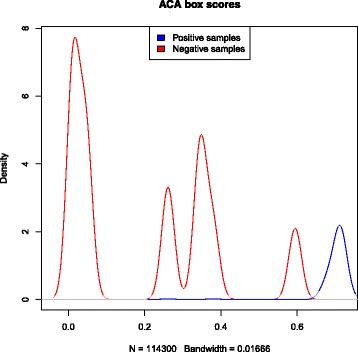
Density plot of *ACA box* PWM-based scores

**Fig. 5:**
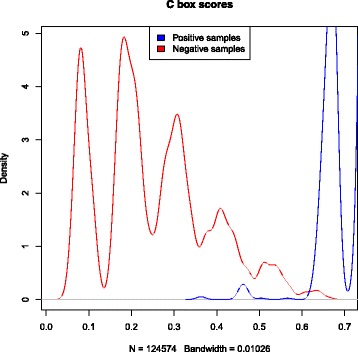
Density plot of *C box* PWM-based scores

**Fig. 6:**
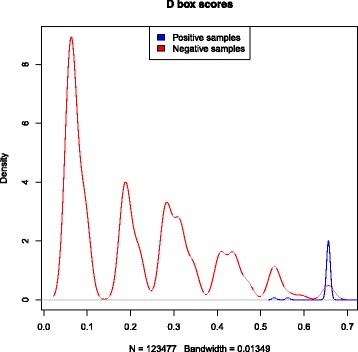
Density plot of *D box* PWM-based scores

Position-specific weight matrices (PWMs) were used to represent each characteristic sequence motif of H/ACA box and C/D box snoRNAs. These PWMs were obtained by scanning the boxes from snoRNAs of vertebrates. A PWM shows the probability that each nucleotide can be found in a particular position of a box motif. These PWMs generate scores used to identify boxes in a candidate sequence. To create thresholds for each box, we scanned snoRNA sequences with a window size equal to the length of the corresponding box. The scanned candidate boxes that were not true boxes were classified as negative boxes. Thus, we generated a density plot to define the thresholds. Figures 3, 4, 5 and 6 show these density plots.

In the validation phase, we used sets of predicted, and partially confirmed with experiments, snoRNAs from many organisms: human [10], nematodes [20], Drosophilids [21], chicken [22], platypus [23] and leishmania [24]. These sequences were manually extracted from Additional files 1, 2 and 3 of each paper (originally in *pdf* format and *doc* format tables).

### Software components

RNA secondary structure prediction was performed using Vienna RNA Package, current version 2.15, in particular RNAfold [25], RNAz [26] and RNALfold [27]. RNAfold predicts a secondary structure associated with the minimum free energy (MFE) of a single stranded RNA or DNA sequence. RNALfold computes locally stable RNA secondary structure with a maximal base pair span. It was used here in order to find the start position of a H/ACA box snoRNA candidate. RNAz was executed to calculate *zscore*, an attribute of the feature vector of H/ACA box snoRNA that represents the thermodynamic stability of a ncRNA secondary structure.

Many tools available in the libSVM version 3.20 [28] performed the classification of H/ACA box snoRNA and C/D box snoRNA:

- *grid.py*: to identify good values for *C* and *γ* SVM parameters;
- *svm-scale*: to scale the feature vector;
- *svm-train*: to perform training and build a model used for predicting new candidates in the *svm-predict* tool;
- *svm-predict*: to predict sequences not used in the training phase.

In order to calculate different performance measures (not available in libSVM), we developed a script using scikit-learn library [29] to calculate Accuracy, F-score, Average Precision, ROC AUC score and Residual sum of squares (RSS). Using these software components, the snoReport 2.0 was entirely rewritten in the C language.

### Identifying snoRNA candidates in genomic sequences

As said before, both classes of snoRNAs, H/ACA box and C/D box, can be distinguished by their characteristic *boxes*, and some specific secondary structure features. For this, each class of snoRNA has a specific way to searching for candidates, described as follows.

**Fig. 7:**
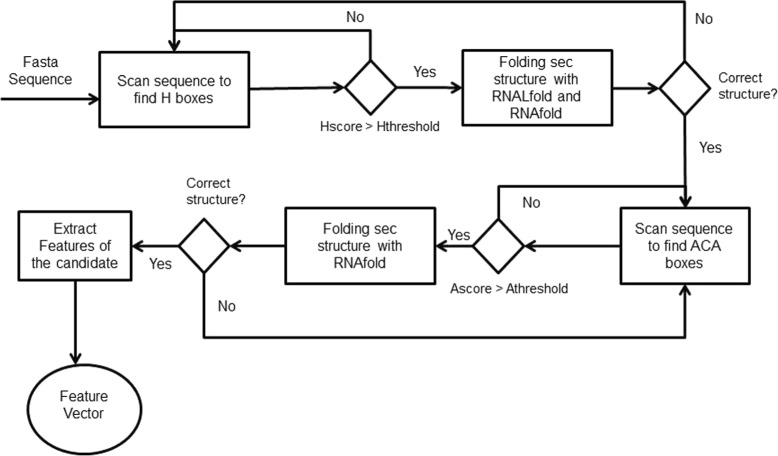
Workflow to identify *H/ACA* on snoReport 2.0
- If one H box candidate has a good PWM-based score, we executed first RNALFold to find the start position of one H/ACA box snoRNA candidate, and then RNAfold with some constraints to predict its secondary structure;
- If the sequence between the start position and the H box candidate has a correct secondary structure, we look for ACA box candidates with a maximum distance of 120 nts and presenting a PWM-based score above a certain threshold;
- Finally, RNAfold is called for the sequence between H box and ACA box. If this sequence has the correct structure, features for this candidate were extracted.

**Fig. 8:**
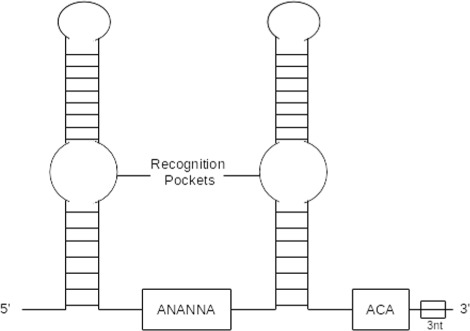
Canonical secondary structure of *H/ACA* box snoRNA, with two hairpins and two short-single stranded regions containing boxes *H* and *ACA* (located 3 nt upstream of the 3’ end). The hairpin contains bulges, or recognition loops, which form complex pseudoknots with the target RNA, where the target uridine is the first unpaired base [7, 10]

Searching for H/ACA box snoRNAs in a genome sequence was performed with the following steps (Fig. 7):

- The genome sequence is scanned in order to find potential H boxes with PWM-based scores above a certain threshold;

Restrictions used to predict secondary structure are specific for each class of snoRNA. For the secondary structure of H/ACA box snoRNA, the region upstream of box H and the region between box H and ACA are used to fold into single stem loop structures. In the cell, snoRNA interacts with a set of different proteins that stabilize the large interior loop containing the target binding site. Without these proteins, standard MFE folding algorithms can predict base pairs within this loop. Therefore, to open the target region, we constrained the 14^*th*^ base upstream of boxes H and ACA, and in most cases the complete interior loop turns out to be unpaired in the MFE structure. Figure 8 shows the canonical representation of H/ACA box snoRNAs.

**Fig. 9:**
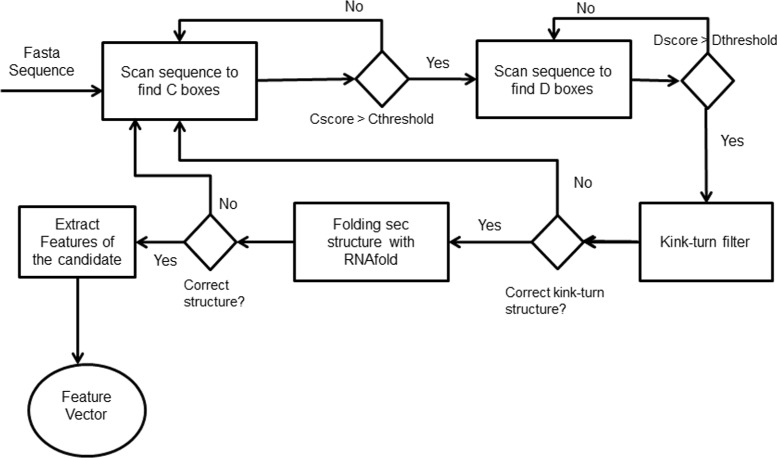
Workflow to identify C/D on snoReport 2.0
- If the C box candidate has a good PWM-based score, we look for D box candidates with a maximum distance of 200 nts with PWM-based score above a certain threshold;
- The candidate has its kink-turn structure (kink turn is a structural motif of RNAs that generates a kink in the helical axis [30]) tested, and in case of having the correct one, RNAfold is called to predict its secondary structure;
- If it has the correct secondary structure, features for this candidate are extracted.

**Fig. 10:**
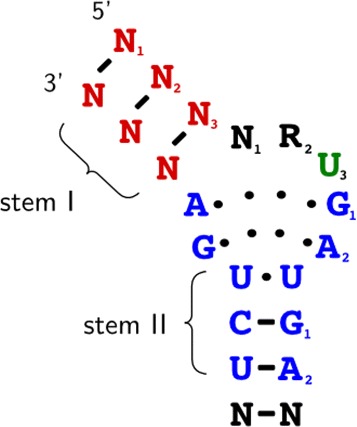
Kink turn structure of *C/D box* snoRNA [31]

**Fig. 11:**
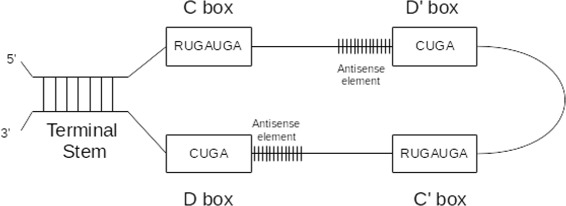
Canonical secondary structure of *C/D box* snoRNA [10]. *Boxes C* and *D* are located near to the 5’ and 3’ ends, noting that they are frequently folded together by a short stem. Normally, imperfect copies of *C* and *D boxes*, called D’ and C’, are located internally in the loop, ordered as C, D’, C’ and D. The target RNA is guided by antisense elements located upstream of *D box* or *D’ box*

Searching for C/D box snoRNAs in a genome sequence was performed with the following steps (Fig. 9):

- The genome sequence is scanned in order to find C boxes with PWM-based scores above a certain threshold;

For the secondary structure of C/D box snoRNA, the complete region from the start of box C to the end of box D has to remain unpaired. Many studies have shown that C/D box snoRNAs must have a perfect kink turn structure that boxes C and D [31–33]. For this, *snoreport* 2.0 has a kink turn structure test, where a C/D box snoRNA candidate must have: G ∙A dinucleotides in box C (RU**GA**UGA) and box D(CU**GA**); at least one uridine on the U-U pair (RUGA**U**GA and C**U**GA); and a Watson-Crick base pair between the 6th nt of C and the 1st nt of D box (RUGAU**G**A and **C**UGA). Figure 10 shows the kink turn structure of C/D box snoRNA, and Fig. 11 shows the canonical representation of C/D box snoRNAs.

#### Extraction of feature vectors

If a snoRNA candidate meets all the previously described filters, and fold the secondary structure, snoReport 2.0 extracts some attributes from a H/ACA (C/D) box snoRNA candidate, in order to build a feature vector, which will be the input for the Support Vector Machine (SVM). Some changes in the feature vectors of both H/ACA box and C/D box snoRNA candidates were introduced, compared to the previous version of snoReport.

In the feature vector of H/ACA box snoRNA, the following new attributes were included: *AC*, *GU*, *zscore*, *Hscore*, *ACAscore*, *LloopSC*, *RloopSC*, *LloopYC*, *RloopYC*, *LloopSym* and *RloopSym*. Table 2 shows all the attributes that have to be extracted from a H/ACA box snoRNA candidate.

**Table 2:** Attributes extracted from a H/ACA box snoRNA candidate

*mfeC*	MFE of the secondary structure with restrictions
	in RNAfold
*A* *C*,*G* *U*,*GC*	AC, GU and GC content
*zscore*	zscore computed by RNAz
*Hscore*	Score of the H box
*ACAscore*	Score of the ACA box
*LseqSize*	Number of nucleotides before the H box
*RseqSize*	Number of nucleotides between H and ACA boxes
*LloopSC*	Lenght of the loop, where we find the pocket region
	containing the target region, near to the H box
*RloopSC*	Length of the loop, where we find the pocket region
	containing the target region, more close to the
	ACA box
*LloopYC*	Symmetry of the loop containing the pocket region
	near to the H box
*RloopYC*	Symmetry of the loop containing the pocket region
	near to the ACA box
*LloopSym*	Symmetry of all loops before H box
*RloopSym*	Symmetry of all loops before ACA box

The attribute *mfeC* shows the MFE of folding with constraint nucleotides, providing the information of how much “effort” is needed to force the candidate sequence to fit the requested structure, or if the candidate is more stable in another structure. AC, GC and GU contents are used to distinguish ncRNAs from other RNAs. For example, the human genome has approximately 42 % of GC content, but single sequences of miRNAs and H/ACA box snoRNAs have 50 % of average GC content [34]. The *zscore* feature is obtained with RNAz [26], representing the thermodynamic stability of a ncRNA secondary structure. Values *Hscore* and *ACAscore* were computed using PWMs of H box and ACA box, respectively. Attributes *LseqSize*, *RseqSize*, *LloopSC*, *RloopSC*, *LloopYC*, *RloopYC*, *LloopSym* and *RloopSym* help to discriminate arbitrary double stem loop structures from H/ACA stem loop structures.

In the feature vector of C/D box snoRNA, new attributes were also included: *zscore*, *bpStem*, *l* *u*5,*l* *u*3, *stemUnpCbox*, *stemUnpDbox*. Table 3 shows the attributes that have to be extracted from a C/D box snoRNA candidate.

**Table 3:** Attributes extracted from a C/D box snoRNA candidate

*mfe*	MFE of the secondary structure without restrictions
	in RNAfold
*mfeC*	MFE of the secondary structure with restrictions
	in RNAfold
*E*_*avg*_	MFE average
*E*_*stdv*_	MFE tandard deviation
*ls*	Length of the terminal stem
*Dcd*	Distance between C and D boxes
*C*_*score*_	score of the C box
*D*_*score*_	score of the D box
*GC*	GC content
*zscore*	zscore obtained by RNAz
*bpStem*	Number of base pairs on the terminal stem
*l* *u*5	Number of unpaired nucleotides inside the stem
	before C box
*l* *u*3	Number of unpaired nucleotides inside the stem
	after D box
*stemUnpCbox*	Number of unpaired nucleotides between the stem
	and the C box
*stemUnpDbox*	Number of unpaired nucleotides between the D box
	and the stem

Attributes *mfeC* and *mfe* are used to distinguish both RNAfold folding procedures, with and without restrictions, respectively. Attributes *E*_*avg*_ and *E*_*stdv*_ represent average and standard deviation of folding energy for random sequences with identical nucleotide frequency in RNAz. Values *Cscore* and *Dscore* were computed using PWMs of C box and D box, respectively. The other attributes (*bpStem*, *l* *u*5, *l* *u*3, *stemUnpCbox*, *stemUnpDbox*) allow to distinguish C/D box snoRNAs from other RNAs according to the stem found by the secondary structure prediction.

#### Training and test phases

**Fig. 12:**
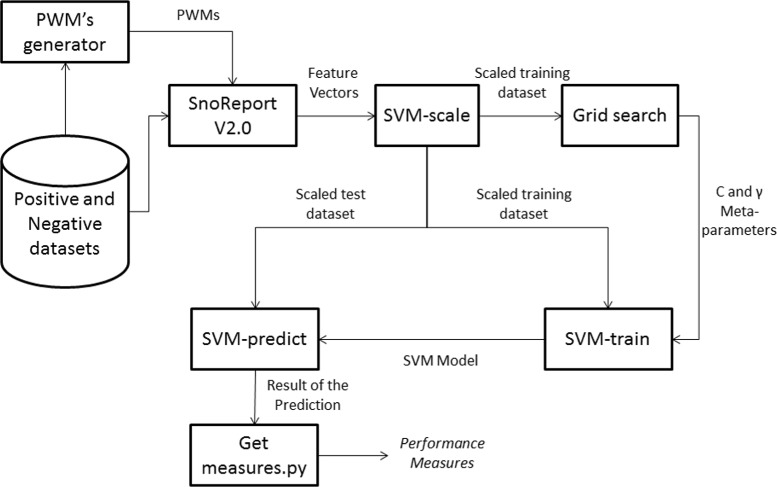
Workflow of snoReport 2.0

Figure 12 shows the training and test phases workflow of snoReport 2.0.

Since we have two datasets for each class of snoRNA, two different training and test phases were performed, one with dataset 1 as training and dataset 2 as test, and vice versa. For each dataset, negative samples were generated with a dinucleotide shuffling procedure from EDeN. In order to reliably measure the quality of the learning, we repeated the training and test phase 10 times for each dataset, generating on each time new negative samples. After creating the training and test dataset, the feature vector was scaled from -1 to 1, using *svm-scale* for a better SVM classification.

**Fig. 13:**
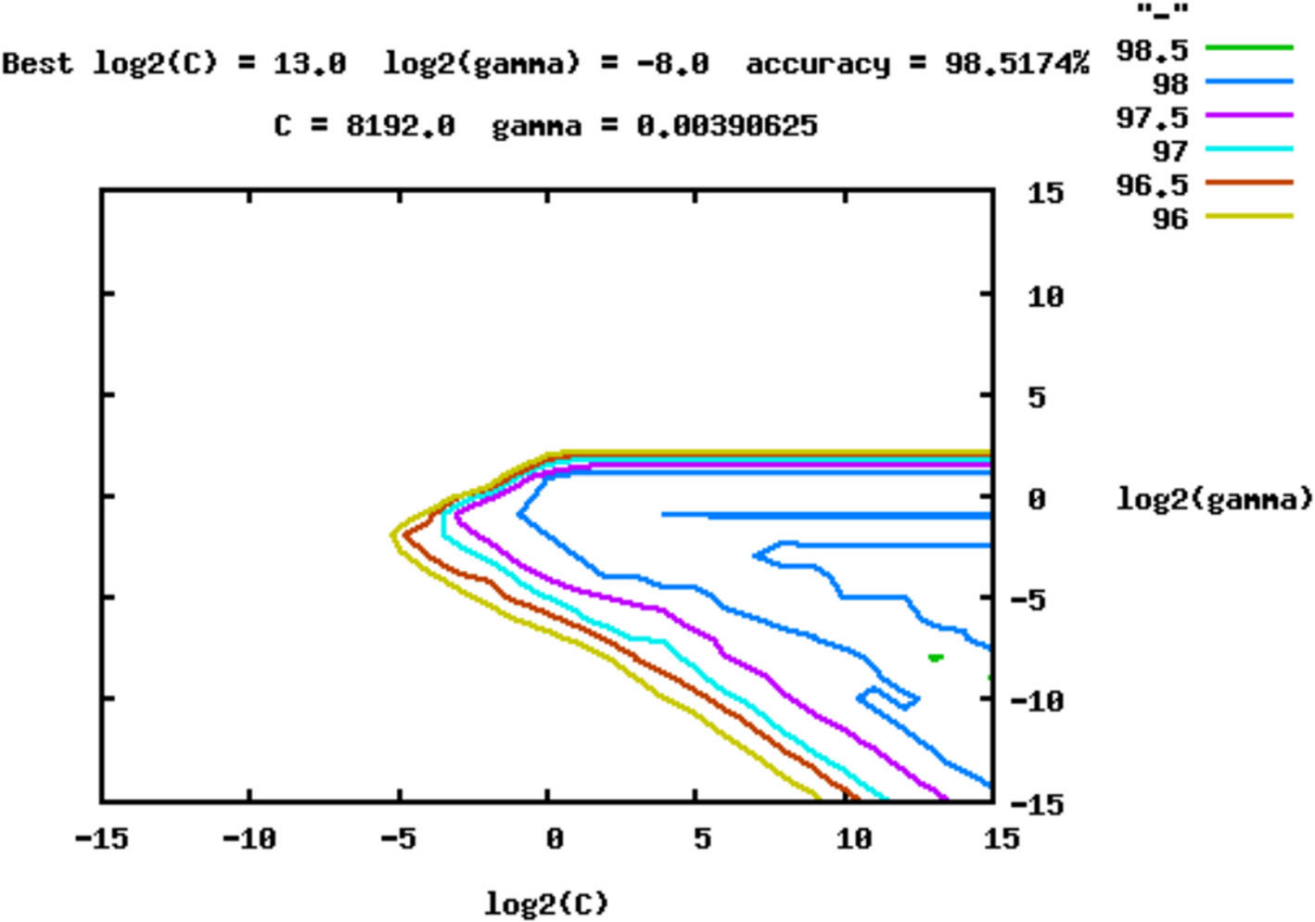
Grid search using accuracy as a criterion for the *C/D box* snoRNA classification. Each line represents the accuracy obtained in the training phase, using parameters *C* and *γ* with 10-fold cross validation. Here, the *green line* represents 98.5 % of accuracy using any point of this line

The next step was to perform a grid search for the *C* and *γ* parameters, using *grid.py* (available in libSVM v3.20), a parameter selection tool for C-SVM classification that uses the RBF (radial basis function) kernel. It uses a cross validation technique (in our case, 10-fold) to estimate the accuracy (another criteria could be used as well) of each combination of C and *γ* in the specified range, which allowed to choose the best values. Following Hsu [35], “a practical method to identify good parameters is to try exponentially growing sequences of C and *γ*”. Therefore, we first investigated all the combinations of these two parameters ranging both from 2^−15^ to 2^15^, shifting 2^1^ for each step of the grid-search (for example, 2^−15^,2^−14^,...2^15^). Figure 13 shows an example of a performed grid search.

After estimating parameters *C* and *γ*, the training phase was performed using *svm-train*, which used C-SVM with the RBF kernel and probabilities estimates enabled. After training, we obtained a classifier (called model) used as input in *svm-predict* to predict snoRNAs from sequences not used in the training phase.

For a more refined analysis, we used the scikit-learn library [29], which allowed to obtain three performance measures to better evaluate and compare the snoReport 2.0 with the previous snoReport:‘q

- Fixed threshold (Accuracy and F-score): a sample is classified as positive if its score (or probability) is above a certain fixed threshold;
- Dynamic threshold (Average precision – APR – and Area Under the Curve – AUC): measures based on moving thresholds along the positive class. It returns the area under the precision-recall curve (APR) and the area under the ROC curve (AUC);
- Residual sum of squares (RSS): shows the discrepancy between data and an the estimator model.

## Results

First, we present statistics of the performance tests. Then we will discuss the results of executing snoReport 2.0 on real data of different organisms.

### Statistics

To identify H/ACA box and C/D box snoRNAs, we built two different datasets for each class of snoRNAs. For the learning phases, we used one dataset as training and the other for test (and vice versa). Each training was repeated 10 times, and our results show the average of the obtained results, together with their corresponding standard deviation. Tables 4 and 5 show the results of the test phase of each snoRNA class obtained with snoReport 2.0.

**Table 4:** Test phase results for H/ACA box snoRNAs: accuracy (Acc), F-score (F-SC), Average Precision (APR), Area under the ROC curve (AUC) and Residual Sum of Squares (RSS). Dat1 and Dat2 means Dataset 1 and Dataset 2, respectively, and SD means *standard deviation*

	Acc (%)	FSC (%)	APR (%)	AUC (%)	RSS
Dat1 → Dat2 average	97.31	93.07	97.85	98.94	0.022
Standard deviation	0.24	0.60	0.20	0.20	0.002
Dat2 →Dat1 average	97.43	94.71	98.66	99.33	98.66
Standard deviation	0.51	1.06	0.42	0.20	0.004
All trainings’ average	97.37	93.89	98.25	99.14	0.021
All training’ SD	0.39	1.19	0.53	0.28	0.003

**Table 5:** Test phase results for C/D box snoRNA. accuracy (Acc), F-score (F-SC), Average Precision (APR), Area under the ROC curve (AUC) and Residual Sum of Squares (RSS). Dat1 and Dat2 means Dataset 1 and Dataset 2, respectively, and SD means standard deviation

	Acc (%)	FSC (%)	APR (%)	AUC (%)	RSS
Dat1 → Dat2 average	94.37	93.67	98.43	98.82	0.044
Standard deviation	1.65	2.04	0.77	0.51	0.012
Dat2 → Dat1 average	96.19	94.94	98.80	99.11	0.029
Standard deviation	0.90	1.25	0.53	0.63	0.007
All trainings’ average	95.28	94.30	98.61	98.96	0.037
All trainings’ SD	1.60	1.77	0.67	0.58	0.012

In order to compare the results with the previous version of snoReport, we executed on snoReport 1.0 the datasets used in the tests with snoReport 2.0. Tables 6 and 7 show the results. These results have shown that snoReport 2.0 presented a better performance to predict vertebrate data, with all the performance measures above 90 %. For H/ACA box snoRNA, the F-score, which consider both precision and recall, snoReport 2.0 was 10.9 % better, having improved the old version. For C/D box snoRNA, we again see an increase of 14,92 % on F-score, and better performances on all the other measures. Thus, snoReport 2.0 showed a significant improvement compared to the previous version.

**Table 6:** Results of the old version of snoReport for H/ACA box snoRNAs using the same datasets used as test on the new version, where: accuracy (Acc), F-score (F-SC), Average Precision (APR), Area under the ROC curve (AUC) and Residual Sum of Squares (RSS). Dat1 and Dat2 means Dataset 1 and Dataset 2, respectively, and SD means *standard deviation*

	Acc (%)	FSC (%)	APR (%)	AUC (%)	RSS
Dat2	92.71	80.62	94.42	96.33	94.42
Standard deviation	0.59	1.23	1.66	0.37	0.004
Dat1	93.31	85.36	95.61	97.37	0.054
tandard deviation	0.25	0.47	0.86	0.28	0.002
All trainings’ average	93.02	82.99	95.01	96.85	0.055
All training’ SD	0.53	2.61	1.42	0.63	0.003

**Table 7:** Results of the old version of snoReport for C/D box snoRNAs using the same datasets used as test on the new version, where: accuracy (Acc), F-score (F-SC), Average Precision (APR), Area under the ROC curve (AUC) and Residual Sum of Squares (RSS). Dat1 and Dat2 means Dataset 1 and Dataset 2, respectively, and SD means *standard deviation*

	Acc (%)	FSC (%)	APR (%)	AUC (%)	RSS
Dat2	90.81	78.27	92.36	96.38	0.076
Standard deviation	0.40	0.73	1.56	0.68	0.003
Dat1	88.67	80.49	96.61	97.79	0.088
Standard deviation	0.25	0.35	0.74	0.42	0.002
All trainings’ average	89.74	79.38	94,49	97.09	0.082
All trainings’ SD	1.15	1.27	2.48	0.91	0.007

### Validation on real data

To verify the quality of prediction, validation on real data was performed with two experiments. In the first one, we executed snoReport 2.0 with a set of previously predicted vertebrate and invertebrate sequences, some of them partially confirmed in experiments, in humans, nematodes, drosophilids, platypus, chickens and leishmania. Tables 8 and 9 show the summary of these results in vertebrates and invertebrates organisms, respectively.

**Table 8:** Results of executing snoReport 2.0 with snoRNA sequences of vertebrate organisms. The number of predicted candidates compared to the number of candidates identified in the cited references are shown

Human		
Yang et al. [10]	C/D: 21/21	H/ACA: 28/32
Platypus		
Schmitz et al. [23]	C/D: 42/144	H/ACA: 45/73
Chicken		
Shao *et al* [22]	C/D: 112/132	H/ACA: 66/69

**Table 9:** Results of executing snoReport 2.0 with snoRNA sequences of invertebrate organisms. The number of predicted candidates compared to the number of candidates identified in the cited references are shown

Nematodes		
Zemann et al. [20]	C/D: 32/108	H/ACA: 46/60
Drosophilids		
Huang et al. [21]	C/D: 2/63	H/ACA: 39/56
Leishmania		
Liang et al. [24]	C/D: 0/62	H/ACA *A-like*: 0/37

Yang et al. [10] identified 54 snoRNAs, 21 C/D box and 32 H/ACA box in human, using snoSeeker, a method based on probabilistic models, pairwise whole-genome alignments of eukaryotes, in which the user can include information of the putative target region or not (to find orphan snoRNAs). The previous version of snoReport predicted 11 out of 21 C/D box snoRNAs and 23 out of 32 H/ACA box snoRNAs, while snoReport 2.0 predicted 21 C/D box snoRNAs and 28 H/ACA box snoRNAs.

Schmitz et al. [23] identified 166 individual snoRNAs in a platypus brain cDNA library, generated from small non-protein-coding RNAs. After, using BLAST searches in platypus genomic sequences, they found 51 more sequences of snoRNA. We predicted 42 out of 144 C/D box snoRNAs, and 45 out of 73 H/ACA box snoRNAs.

Shao et al. [22] identified 132 C/D box snoRNAs in chicken using *CDseeker* and 69 H/ACA box snoRNAs using *ACAseeker* (both programs are used in snoSeeker [10]). We predicted, with snoReport 2.0, 112 out of 132 C/D box snoRNAs, and 66 out of 69 H/ACA box snoRNAs.

Zemann et al. [20] used a combination of high-throughput cDNA library screening and computational search strategies to find 121 snoRNAs (168 are shown in their supplementary material) in *Caernorhabditis elegans*. Our snoReport 2.0 predicted 32 out of 108 C/D box snoRNAs, and 46 out of 60 H/ACA box snoRNAs.

Huang et al. [21] performed a large-scale genome wide analysis to identify both classes of snoRNAs in *Drosophila melanogaster* using experimental and computational RNomics methods, having found 119 snoRNAs. Our snoReport 2.0 predicted 2 out of 63 C/D box snoRNAs, and 39 out of 56 H/ACA box snoRNAs.

Finally, Liang et al. [24] used a genome-wide screening approach to identify 62 C/D box snoRNAs and 37 H/ACA box snoRNAs of closely related pathogens of *Leishmania major*. We did not identify any C/D box nor H/ACA box snoRNAs. It is interesting to note that H/ACA box snoRNAs from *Leishmania major* are quite different from the canonical H/ACA box snoRNAs of yeast and vertebrate. For example, they lack a recognizable H box, presenting an AGA box instead of an ACA box [9]. Our snoReport 2.0 was designed to identify canonical snoRNAs from many different organisms, thus to predict H/ACA box snoRNAs from organisms that are different from the canonical model, we should use a different training set, together with a revision of the attributes of the feature vector.

In the second experiment, we investigated false positives in snoReport 2.0. A variety of ncRNA families were taken from RFAM [36] (100 sequences with sizes compatible to snoRNAs), and a set of 100 randomly chosen genomic *loci* of snoRNA comparable size taken from human genome GRCh38.p7 [37] chromosomes 15, 16, 21 and 24 (25 sequences from each chromosome). To construct the confusion matrices (Tables 10 and 11), we chose one representative sequence from each cluster of snoRNAs, as described in the data source section, in a total of 224 snoRNAs, 132 C/D box and 92 H/ACA box.

**Table 10:** Confusion Matrix of C/D box snoRNA validation experiment using real data

	Predicted as	Predicted as
	C/D box	non C/D box
C/D box snoRNAs (132)	98	34
Non C/D box snoRNAs (200)	0	200

**Table 11:** Confusion Matrix of H/ACA box snoRNA validation experiment using real data

	Predicted as	Predicted as
	H/ACA box	non H/ACA box
H/ACA box snoRNAs (92)	60	32
Non H/ACA box snoRNAs (200)	4	196

For the C/D box snoRNA experiment, we obtained a precision of 100 %, a recall of 74.2 % and a F-score of 85.2 %. This shows that snoReport 2.0 is reliable to predict true C/D box snoRNAs, since no other kind of ncRNA was predicted as C/D box snoRNA.

For the H/ACA box experiment, we obtained a precision of 93.8 %, a recall of 65.2 % and a F-score of 76.9 %. Analogous to the C/D box snoRNA experiment, our method prevent to obtain false positives, confirming that it is reliable to predict snoRNAs. Regarding the four non H/ACA box snoRNAs identified as so, three sequences belonging to chromosome 15 and one to chromosome 16. It is noteworthy that, in chromosome 15, one unknown H/ACA box snoRNA, with probability of 93 %, was located inside the protein TRPM1 [38]. The other two snoRNAs were located in uncharacterized contigs. In chromossome 16, we identified a H/ACA box snoRNA, with probability of 91 %, inside the uncharacterized LOC102723323 ncRNA [39].

## Discussion

In this work, we refined the training phase of the SVM method, using different features in the characteristic vector, more data from different vertebrate organisms, and new versions of the tools and data bases used to build the first version of snoReport. We carefully chose good values for the *C* and *γ* SVM parameters using grid searches.

All these steps allowed us to improve the performance of snoReport, avoiding false positives and finding more snoRNAs. H/ACA box snoRNA classifier had an improvement of 10.9 % regarding to F-score, with the same data, when compared to the first version of snoReport. Besides, the high score achieved from average precision,ROC AUC score and RSS show us that the predictions have a high degree of reliability. The same could be observed for C/D box snoRNA classifier, which have an improvement of 14.92 % regarding to F-score, and more than 90 % of all performance measures presented, allowing us to have high rate of quality on each prediction.

The validation phase showed, in the first experiment, that snoReport 2.0 predicted 67.43 % of snoRNAs from vertebrates organisms, which shows that snoReport 2.0 can identify snoRNAs with significantly higher precision while maintaining recall. It is noteworthy that many sequences used for validation was not yet experimentally validated, and maybe some of them can be false positives, or are not representatives of the canonical snoRNAs (like the snoRNAs in leishmania). In this case, snoReport 2.0 could discard these candidates. Since snoReport was trained with vertebrate sequence, snoRNAs in invertebrates could not be detected efficiently by snoReport. To deal with some of these organisms, it is necessary to discover new features that describe those non standard snoRNAs and use particular datasets in machine learning tasks. However, we find 69,64 % and 76,67 % of H/ACA box snoRNAs of nematodes and drosophilids described in literature, which suggests that H/ACA box snoRNA predictor from snoReport can be used with high performance. In the second experiment, the validation confirmed that snoReport 2.0 prevents to prediction of false positives.

Therefore, snoReport 2.0 constitutes a substantial improvent over its first version, and is now more efficient and reliable to identify both classes of snoRNAs. It can be used for many different organisms, even invertebrates, with high quality of prediction.

## Conclusion

In this article, we presented snoReport 2.0, a reliable and efficient tool to predict the two main classes of snoRNAs in different organisms. This version is a refinement of a previous version of snoReport, obtained with extensive improvements in the SVM method, and the use of new versions of tools (specially those to predict secondary structures) and databases. In contrast to previous methods for snoRNA identification, snoReport 2.0 can identify both guide and orphan snoRNAs without using any information of putative target sites within ribosomal or spliceosomal RNA nor using multiple alignments. Experiments with very different organisms have shown good performance, even in invertebrates organisms (for H/ACA box snoRNA), showing that snoReport 2.0 can be used to obtain reliable prediction of snoRNAs in a variety of organisms. Besides, it prevents to predict false positives.

Future work include to create specific datasets for different kinds of organisms (e.g, for invertebrates), and to study at what extent different approaches to fold the sequences and different machine learning methods (e.g., using EDeN to transform the secondary structure of snoRNAs in a graph representation, that can be decomposed in a sparse vector) allow to find intrinsic features or even to predict new snoRNAs. Clearly, these techniques could affect the performance of snoReport 2.0. Our method could also be used to identify snoRNAs in specific species, e.g., fungi (*Paracoccidioides brasiliensis*, *Schizosaccharomyces pombe* and *Pichia pastoris*), or to find specific features and perform a SVM training to identify snoRNAs in leishmania. Finally, a general method could be developed to allow SVM training with particular organisms, according to an user’s necessity.

## Availability and requirements

- **Project Name**: SnoReport v2.0;
- **Project home page**: http://www.biomol.unb.br/snoreport;
- **Operation system(s)** Linux;
- **Programming language** C ansi;
- **Other requirements**: Vienna RNA Package v2.1.5 (particularly RNAfold, RNALFold and RNAz);
- **License**: GNU GPL
- **Any restriction to use by non-academics**: No restrictions

## Additional files

Additional file 1 Trees of C/D box snoRNAs and H/ACA box snoRNAs. The generated distance trees of C/D box snoRNA and H/ACA box snoRNA clusters, used to build the datasets for the training and testing phases. (PDF 47 kb)

Additional file 2 PWMs to identify snoRNA boxes. Position-specific weight matrices (PWMs) used to identify boxes of both classes of snoRNAs. (PDF 64 kb)

Additional file 3 Machine learning statistics. Statistics of all learning procedures made. (ODS 61 kb)

## Abbreviations

MFE: Minimum free energy
ncRNA: Non-coding RNA
PWMs: Position-specific weight matrices
snoRNA: Small nucleolar RNA
SVM: Support Vector Machine
