# Supplementary figures and images for: SnoReport 2.0: new features and a refined Support Vector Machine to improve snoRNA identification
Source: BMC Bioinformatics. 2016 Dec 15;17(Suppl 18):464. doi: 10.1186/s12859-016-1345-6 (PMC5249026; doi:10.1186/s12859-016-1345-6)

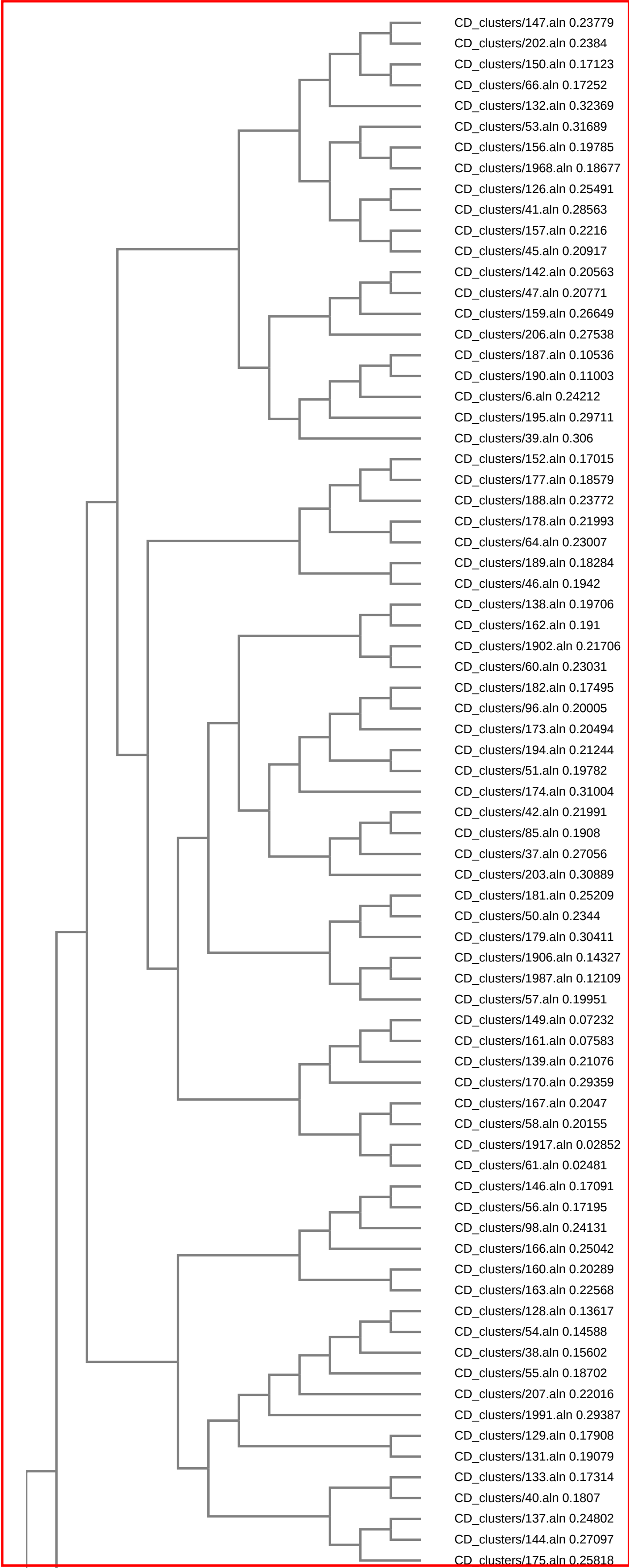

Dataset 1

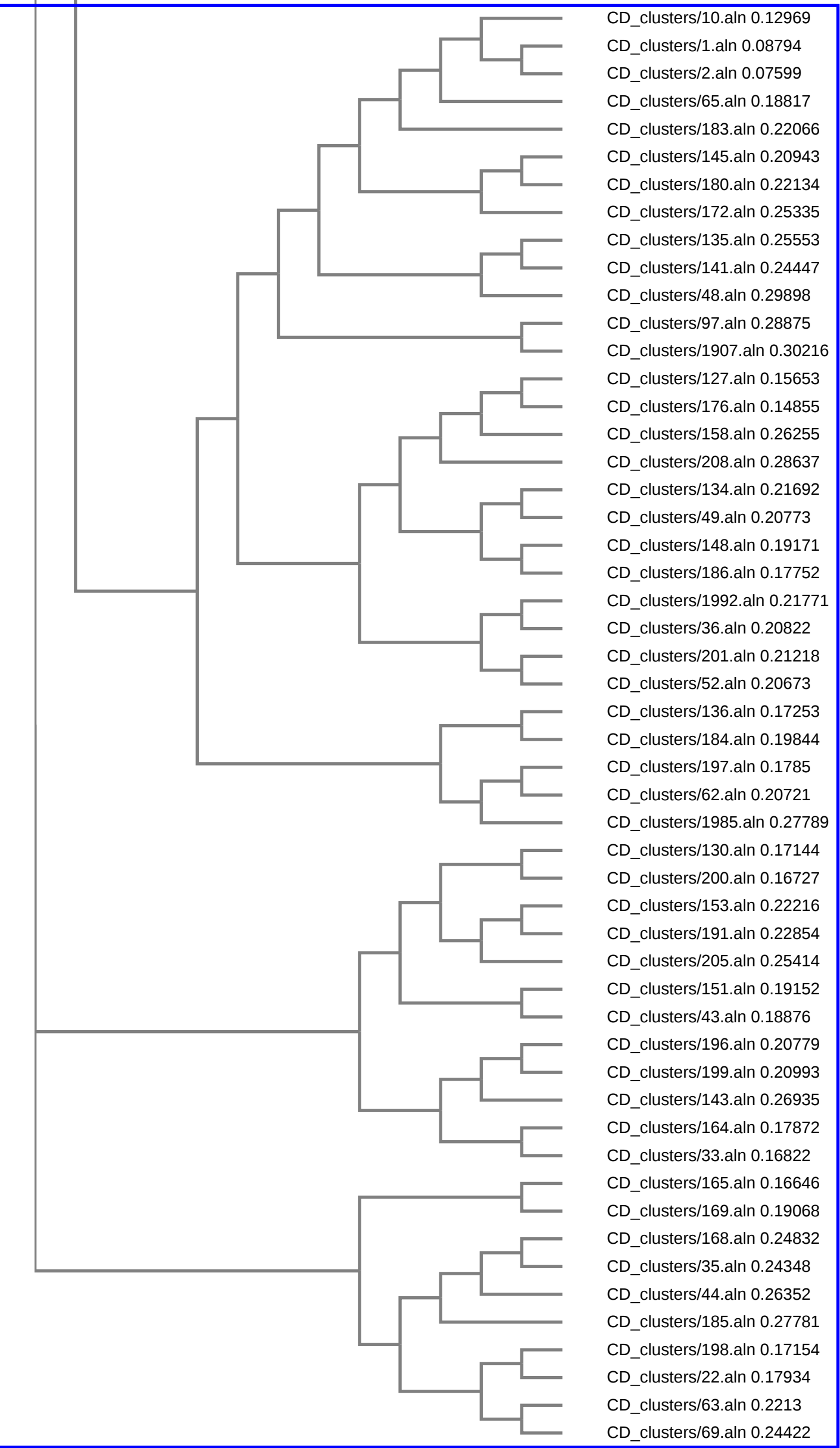

Dataset 2

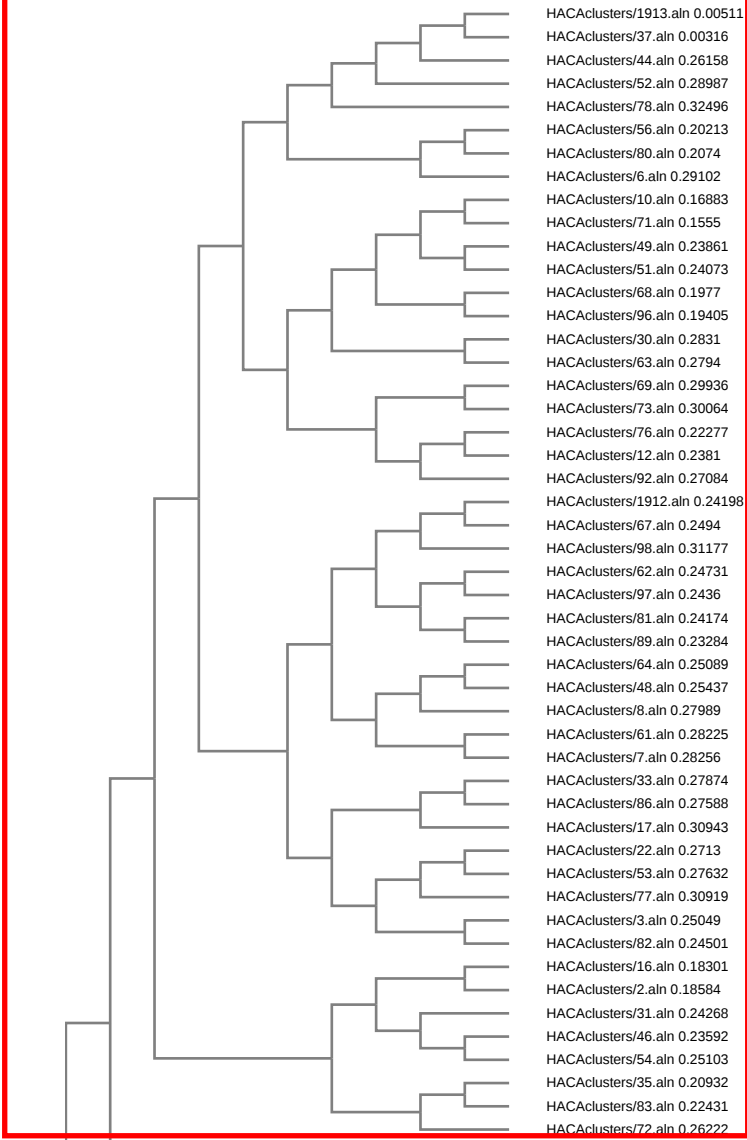

Dataset 1

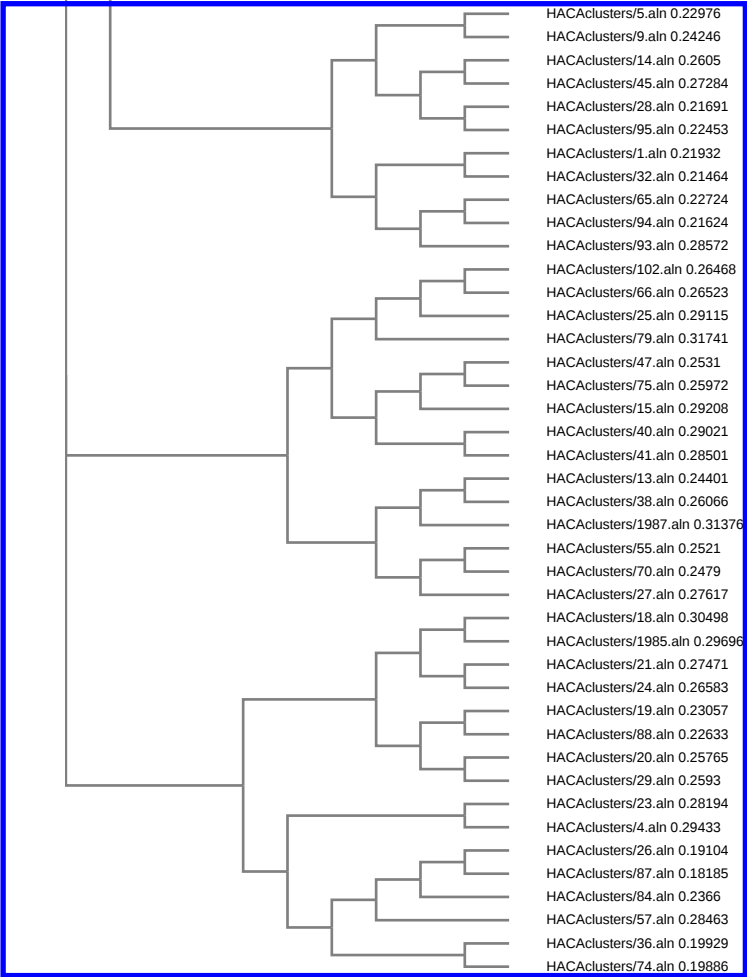

Dataset 2

Supplement: Additional file 1 — Trees of C/D box snoRNAs and H/ACA box snoRNAs. The generated distance trees of C/D box snoRNA and H/ACA box snoRNA clusters, used to build the datasets for the training and testing phases. (PDF 47 kb) [file 12859_2016_1345_MOESM1_ESM.pdf]
