# Supplementary material for: SnoReport 2.0: new features and a refined Support Vector Machine to improve snoRNA identification
Source: BMC Bioinformatics. 2016 Dec 15;17(Suppl 18):464. doi: 10.1186/s12859-016-1345-6 (PMC5249026; doi:10.1186/s12859-016-1345-6)
Supplement: Additional file 2 — PWMs to identify snoRNA boxes. Position-specific weight matrices (PWMs) used to identify boxes of both classes of snoRNAs. (PDF 64 kb) [file 12859_2016_1345_MOESM2_ESM.pdf]

PWMs of C, D, H and ACA boxes

João Victor de Araujo Oliveira[1]

[1]Department of Computer Science, University of Brasilia, Brazil

May 30, 2016

C box PWMs

Table : C box PWMs

A	C	G	U
0.3922	0.1569	0.2549	0.1961
0.0784	0.0980	0.0588	0.7647
0.0000	0.0392	0.9412	0.0196
0.9608	0.0196	0.0000	0.0196
0.0588	0.0784	0.1176	0.7451
0.0980	0.0000	0.8235	0.0784
0.7451	0.0588	0.1176	0.0784

D box PWMs

Table : D box PWMs

A	C	G	U
0.1250	0.6250	0.1250	0.1250
0.1250	0.1250	0.1250	0.6250
0.0000	0.0000	1.0000	0.0000
0.8750	0.1250	0.0000	0.0000

H box PWMs

Table : H box PWMs

A	C	G	U
0.9558	0.0128	0.0223	0.0091
0.25	0.25	0.25	0.25
0.9774	0.0057	0.0134	0.0035
0.25	0.25	0.25	0.25
0.25	0.25	0.25	0.25
0.8025	0.0472	0.1216	0.0287

ACA box PWMs

Table : ACA box PWMs

A	C	G	U
0.9999	0.0000	0.0001	0.0000
0.0448	0.7840	0.0315	0.1396
0.9998	0.0002	0.0000	0.0000
